# Prevalence of obesity and overweight and its associated factors among registered pensioners in Ghana; a cross sectional studies

**DOI:** 10.1186/s40608-017-0162-4

**Published:** 2017-07-04

**Authors:** Benjamin D. Nuertey, Alabira Iddrisu Alhassan, Augustine D. Nuertey, Isaac Asimadu Mensah, Victor Adongo, Clement Kabutey, Joyce Addai, Richard Bekoe Biritwum

**Affiliations:** 1 Tamale Teaching Hospital, Tamale, Ghana; 2 Korle-Bu teaching Hospital, Korle-Bu, Accra, Ghana; 3 Community health department, School of Public Health, University of Ghana, Korle-bu, Accra, Ghana; 4 West African Center of Cell Biology and Infectious Pathogens, University of Ghana, Legon, Accra, Ghana; 5 St Francis Xavier Hospital, Assin Fosu, Ghana; 6 Nursing and Midwifery training College, Koforidua, Ghana

**Keywords:** Obesity, Overweight, Pensioners, Body mass index

## Abstract

**Background:**

The elderly are faced with health problems such as cardiovascular diseases, type II diabetes mellitus, kidney disease, arthritis and other musculoskeletal problems, which can be linked to obesity and overweight. The aim of this study was to determine the prevalence of obesity and overweight and its associated factors amongst registered pensioners in Ghana.

**Methods:**

A cross-sectional study was conducted among members of the national pensioners association of Ghana. 4813 pensioners took part in the study. Thirteen study centers were used in the study with at least one center per regional capital. Questionnaires, physical examinations, blood and urine sample examinations were carried out.

**Results:**

Overall, 16.3% of the pensioners were obese while 30.0% were overweight. Prevalence of obesity among males and females were 8.0% and 34.5% respectively. Pensioners who were hypertensive had 1.8 times the odds (95% CI = 1.5–2.0) of being obese/overweight. Serum triglycerides levels of 2.26 mmol/L (200 mg/dL) or more, were associated with 80% chance of obesity and overweight (OR = 1.8, 95% CI = 1.3 - 2.5). There was 30% increase in arthritis among obese/overweight pensioners compared to normal/underweight pensioners. Obesity and overweight pensioners were more likely to be using eyeglass (OR = 1.7, 95% CI = 1.3–2.2) and less likely to report hearing loss (OR = 0.7, 95% CI =0.5–1.0).

**Conclusion:**

The prevalence of obesity among the elderly in Ghana is high. Age had an inverse linear relationship with BMI among pensioners. Hypertension, arthritis and dyslipidemia were associated with obesity among registered pensioners.

## Background

The population of pensioners is progressively increasing. In the developing world, the elderly population is seen to represent the fastest growing segment of the population [1]. In the year 2015, Ghana’s population of the elderly aged 60 years and above was 5.3% and it is expected to reach 9.7% by 2050 [2]. The 2010 Global Burden of Disease attributed 23.1% of total disease burden to disorders in people aged 60 years and above and it is expected to increase with population aging [3, 4]. The elderly are faced with social, economic and medical problems with very few elderly living a disease free life [3].

Clinical pathways regarding obesity and its associated factors depict a dual relationship. First obesity as a risk factor for certain disease and secondly as an outcome of several other genetic, behavioral, socioeconomic and environmental factors [5]. As a risk factor, overweight and obesity increases the risk for cardiovascular diseases, type II diabetes mellitus, kidney disease, arthritis and other musculoskeletal problems [6–8]. It also increase the risk for depression, some cancers, insomnia and chronic fatigue [9, 10]. Obesity is associated with deterioration of health related quality of life and increased mortality [11–13]. Epidemiological studies investigating the effect of obesity and overweight on elderly patient survival reports a reverse epidemiology termed as obesity paradox in geriatric population [14–16].

In the year, 2010, the burden of disease attributed to high body mass index was estimated at 94 million disability-adjusted life-years [4]. Despite the problems of obesity in the elderly, health research in the developing countries has been and continues to be heavily centered on younger and working population groups [17]. Globally there is an increasing trend in obesity among all age group [18]. In the year 2008, an estimated 1.4 billion adults were overweight of which about 500 million were obese [4, 5]. Six years after and in the year 2014, more than 1.9 billion adults representing 39% of adults aged 18 years and older were overweight of which over 600 million were obese [9]. It is projected that by the year 2030, there will be 2.16 billion overweight and 1.12 billion obese persons alive globally [19]. The global prevalence of obesity among male and female aged 60–64 year old is estimated at 13 and 19% respectively [20].

A recent systematic review and meta-analysis conducted in Ghana reported a nationwide prevalence of Obesity and overweight as 17.1% and 25.4% respectively [21]. The World Health Organization Study on global Aging and adult health (SAGE), wave one reported a prevalence of obesity or overweight among the elderly as 30% [22]. Prevalence of Obesity and overweight among pensioners in Ghana is however lacking.

The aim of this study is to determine the prevalence of obesity and overweight among the pensioners in Ghana. It also seeks to identify the factors associated with obesity and overweight among the pensioners in Ghana. The study reported on the prevalence of obesity within different strata of the pensioner population. It also reported on several socio-demographic as well as medical factors associated with obesity and overweight.

## Methods

### Study design

A cross sectional study was conducted in all ten regional capitals of Ghana among the members of the National pensioners association (NPA). The survey was conducted as part of a registration exercise for all members of the National Pensioners association for the start of a Pensions Medical Scheme (PMS). It took place from April to December of 2014. All the members of the national pensioners association converge at the registration/medical screening within the days of the screening. The medical screening in each of the regions took place in the regional capitals of the respective regions. Participants were made to rest for at least 15 min, after which they go through the medical screening exercise.

### Study population and eligibility criteria

The study subjects were registered pensioners. Ghana’s mandatory retirement age is 60 years. However, there were some participants below 60 years who have retired before the retirement age of 60 years due to disability and other reasons thereby qualifying as a pensioner. Within the study period, 4813 members of the national pensioners association presented for the medical screening. Participant must be a member of the National Pensioners association and must be resident in Ghana.

### Sample size and sampling method

A census of all the participants within the selected sites was the target. Members of the National pensioners association resident in the regional capital of the respective region and their surrounding towns and villages were eligible.

### Study materials and data capture tool

Study questionnaires were made to record socio-demographic data of participants. It had a section on past medical history collecting information on known past medical history. It further collected information on allergies, alcohol, smoking, exercise and diet. There was a physical examination form attached, which was used to capture data of the physical examination conducted by medical officers. Weight was measured with a weighing scale and height with a stadiometer. The Blood pressure was measured using standardized electronic sphygmomanometer with appropriate cuff sizes. Visual acuity was checked using a Snelling chart. Blood sample was taken and a glucometer was used to measure the random blood sugar. Blood sample was collected into a serum separator bottle and transported to a laboratory for measurement of serum cholesterol using automated and standardized techniques.

### Data collection

Trained research assistants fill the questionnaire based on the response of the study participants. The weight was measured with the weighing scale to the nearest one-kilogram while the height to the nearest one millimeter. Medical officers perform physical examination while the eye team checked the vision and performed fundoscopy. Personalized reports of the medical screening were made available to each participant.

### Data processing and analysis

The data generated in the research were entered into Epidata 3.1 and exported into STATA/MP 11.0 (copyright 2004–2009) for analysis. The primary outcome in the study was overweight and Obesity. With regard to social class, participants previous occupation was classified under various social class heading according to the registrar general’s occupational classification of England and wales. The background characteristics of the respondents were obtained by cross tabulation. Logistic regression was used to analyze the factors associated with obesity. First, the association between each of the potential factors and overweight/obesity was examined ignoring other variables. This analysis was important because it gave a fair idea as to which of the variables were strong predictors/related to overweight and obesity. Second, to construct a model with factors that are independently associated with overweight and obesity, each of the independent variable was a candidate provided that the *p*-value was 0.05 or less. Three models were reported. In model A: religion, region of residence, ethnic background and marital status were locked in the model and therefore adjusted for. Model B adjusted for age, sex, highest formal education, social class and all the variables adjusted for in model A whiles model C adjusted for all variables adjusted for in model A, plus all other variables in the table.

### Ethical considerations

Review and approval was obtained from the National Pensioners Association board. The board further monitored each step of the data collection process. Members of the National Pensioners association gave the consent to participate. The content of the medical screening exercise was developed in extensive consultation with the executives of the National Pensioners Association. Consent was voluntary and each study participant had the right to withdraw at any stage of the study process. Uttermost privacy and confidentiality were maintained. No compensation or payments were made to any study participants. However examination findings were explained to participants. Personalized results of the study were sent to each participant. Data files were password protected. Hard copies were stored in locked file cabinets, and access was limited to the Principal investigator.

## Results

### Background characteristics of study participants

Table 1 displays the background characteristics of the study participants. 4476 of the 4813 members of the national pensioners association that presented at the thirteen centers across the country took part in the study. This translates to an overall response rate of 93.0%. The mean weight of all participants was 66.7Kg (SD 13.2 kg). Females (69.3, SD = 14 Kg) were heavier compared to their male (65.6, SD 12.8) counterparts. The males were however taller compared to the females; the mean height and standard deviations of males and female pensioners were 165.7 CM (SD = 7.6) and 157.2 CM (SD = 7.0) respectively.

**Table 1:** Background characteristics of 4813 study participants

	Participants			Normal/Underweight	Overweight/Obese
Variables	All	Male	Female		
	Mean (SD)	Mean (SD)	Mean (SD)	Mean (SD)	Mean (SD)
Weight (Kg)	66.7 (13.2)	65.6 (12.8)	69.3 (14.0)	58.4 (8.1)	76.4 (11.3)
Height (cm)	163.0 (8.4)	165.7 (7.6)	157.2 (7.0)	164.6 (8.0)	161.2 (8.4)
BMI (Kg/m^2^)	25.2 (5.1)	23.9 (4.3)	28.1 (5.5)	21.5 (2.3)	29.4 (4.0)
Age (years)	67.2 (5.4)	68.0 (5.6)	65.5 (4.4)	68.1 (5.7)	66.2 (4.9)
Blood sugar^b^ (mmol/L)	5.8 (2.8)^a^	5.7 (2.5)^a^	6.0 (2.9)^a^	5.6 (2.2)^a^	6.2 (2.9)^a^
Blood Pressure (mmHg)					
Systolic	146 (26)	146 (25)	146 (26)	144 (27)	149 (25)
Diastolic	79 (16)	79 (16)	79 (16)	77 (16)	81 (16)
Serum Lipids (mmol/L)					
Total Cholesterol	4.90 (1.24)	4.69 (1.24)	5.37 (1.30)	4.74 (1.18)	5.08 (1.29)
Triglycerides	1.21 (0.68)^a^	1.17 (0.64)^a^	1.30 (0.76)^a^	1.14 (0.62)^a^	1.30 (0.75)^a^
LDL-Cholesterol	2.83 (1.05)	2.67 (1.00)	3.18 (1.1)	2.74 (1.00)	2.92 (1.10)
HDL-Cholesterol	1.47 (0.34)	1.44 (0.33)	1.54 (0.36)	1.44 (0.33)	1.51 (0.35)
Coronary risk ratio	3.4 (0.9)	3.3 (0.8)	3.6 (0.9)	3.4 (0.8)	3.5 (0.9)

Mean (SD): Mean and standard deviation provided for all variables except (^a^) where median and interquartile range provided

Median (interquartile range)^a^ is provided if −1.5 ≤ co-efficient of skewness ≥1.5

Blood sugar^b^; Random blood sugar

BMI of the females were higher compared to males: 28.1Kg/M^2^ (SD = 5.5) and 23.9 Kg/M^2^ (SD = 4.3) respectively. The mean age of participants was 67.2 years (SD = 5.4). The males were older compared to females with mean ages of 68.0 (SD 5.60 and 65.5 (SD = 4.4) respectively. Also the obese and overweight group were comparatively younger compared to the normal and underweight group. The mean age of the obese and overweight group was 62.2 (SD = 4.9) while the underweight and normal age group was 68.1 (SD = 5.7).

With regards to serum lipids, the mean total cholesterol for all study participants was 4.90 mmol/L (SD = 1.24). Females as well as obese and overweight participants had higher total cholesterol compared to males. The mean systolic blood pressure, the mean diastolic blood pressure and the mean arterial blood pressure was the same among the male and female participants. Both males and females had mean systolic blood pressure of 146 (SD = 26) mmHg and a mean diastolic blood pressure of 79 (SD = 16) mmHg.

### Prevalence of underweight, normal, overweight and obesity among pensioners in Ghana

Table 2 displays the prevalence of obesity, overweight, normal and underweight among the pensioners in Ghana. Overall, 16.3% of the pensioners were obese while 30.0% were overweight. Majority of the pensioners accounting for 47.7% were within normal BMI weight classification. 6.0% of the participants were underweight as shown in Table 2. Prevalence of obesity was lower among the males pensioners compare to that of the females. Males had an obesity prevalence of 8.0% while the prevalence among the females was 34.5%. Likewise the prevalence of overweight among the females was higher than that among the males accounting for 35.1% and 27.6% respectively. With regard to age, the prevalence of obesity decreases with increasing age. Pensioners aged less than 65 had the highest prevalence of obesity (20.6%) and it decreases among the ages group such that the prevalence of obesity among the above 80 years population is about 3.1%. Similarly, overweight shows a decreasing prevalence relationship with increasing age as shown in Table 2.

**Table 2:** Prevalence of underweight, normal, overweight and Obesity among pensioners in Ghana, stratified by socio-demographic factors

		Body Mass index groupings			
		≤18.4	18.5–24.9	25.0–29.9	≥ 30
Variable		Underweight	Normal	Overweight	Obese
	*N*	*n* (%)	*n* (%)	*n* (%)	*n* (%)
Overall Prevalence	4476	267 (6.0)	2137 (47.7)	1342 (30.0)	730 (16.3)
Prevalence by Sex					
Male	3069	232 (7.6)	1743 (56.8)	848 (27.6)	246 (8.0)
Female	1402	34 (2.4)	393 (28.0)	492 (35.1)	483 (34.5)
Prevalence by Current Age in years					
Less than 65	1625	72 (4.4)	659 (40.6)	559 (34.4)	335 (20.6)
65–69	1484	68 (4.6)	711 (47.9)	453 (30.5)	252 (17.0)
70–74	774	59 (7.6)	425 (54.9)	195 (25.9)	95 (12.3)
75–79	333	36 (10.8)	198 (59.5)	73 (21.9)	26 (7.8)
≥ 80	130	22 (5.9)	71 (54.6)	33 (25.4)	4 (3.1)
Prevalence by Religion					
Christianity	3793	202 (5.3)	1752 (46.2)	1168 (30.8)	671 (17.7)
Islam	476	40 (8.4)	263 (55.3)	129 (27.1)	44 (9.2)
Traditional	90	15 (16.7)	65 (72.2)	9 (10.0)	1 (1.1)
Others	18	1 (5.6)	8 (44.4)	6 (33.3)	3 (16.7)
Prevalence by Highest Formal Educational Level					
None	484	49 (10.1)	289 (59.7)	112 (23.1)	34 (7.0)
Basic/ MSLC^a^	1596	93 (5.8)	797 (49.9)	464 (29.10	242 (25.2)
Secondary	618	34 (5.5)	292 (39.5)	188 (30.4)	104 (16.8)
Tertiary	1141	44 (3.9)	451 (39.5)	398 (34.90	248 (21.7)
Others	163	5 (3.0)	70 (42.9)	57 (35.0)	31 (19.0)
Prevalence by social class^a^					
I [Professional]	309	5 (1.62)	76 (24.6)	111 (35.9)	117 (37.9)
II [Managerial/technical]	780	39 (5.0)	357 (45.8)	248 (31.8)	136 (17.4)
III [(N) Skilled non-manual]	1287	71 (5.5)	557 (43.3)	446 (34.7)	213 (16.6)
III [(M) Skilled Manual]	385	24 (6.1)	222 (56.2)	105 (26.6)	44 (11.1)
IV [Partly skilled]	863	53 (6.1)	449 (52.0)	240 (27.8)	121 (14.0)
V [Unskilled]	682	63 (9.2)	392 (57.5)	157 (23.0)	70 (10.30
Prevalence by Current marital status					
Never Married	73	5 (6.9)	32 (43.8)	25 (34.3)	11 (15.1)
Married	3155	198 (6.3)	1629 (51 .6)	919 (29.2)	409 (13.0)
Widow/Widower	695	36 (5.2)	249 (35.8)	226 (32.5)	184 (26.5)
Divorced	216	7 (3.3)	80 (37.0)	72 (33.3)	57 (26.4)
Separated	181	11 (6.1)	71 (39.2)	59 (32.6)	40 (22.1)
Prevalence by ethnicity					
Ga-Dangme	185	8 (4.3)	70 (37.8)	60 (32.4)	47 (25.4)
Akan	2215	101 (4.6)	975 (44.0)	715 (32.3)	424 (19.1)
Ewe	749	33 (4.4)	349 (46.6)	240 (32.0)	127 (17.0)
Guan	99	6 (6.1)	44 (44.4)	27 (27.3)	22 (22.2)
Mole-Dagomba	832	87 (10.5)	491 (59.0)	197 (23.7)	57 (6.9)
Grusi	84	11 (13.1)	33 (39.3)	26 (31.0)	14 (16.7)
Others	312	21 (6.7)	175 (56.1)	77 (24.7)	39 (12.5)
Prevalence by region of residence					
Ashanti	1045	41 (3.9)	445 (42.6)	353 (33.8)	206 (19.7)
Brong Ahafo	275	16 (5.8)	142 (51.6)	97 (35.3)	20 (7.3)
Central	166	20 (12.1)	100 (60.2)	36 (21.7)	10 (6.0)
Eastern	402	38 (9.5)	207 (51.5)	100 (24.9)	57 (14.2)
Greater Accra	374	9 (2.4)	164 (43.9)	107 (28.6)	94 (25.1)
Northern	329	17 (5.2)	167 (50.8)	105 (31.9)	40 (12.2)
Upper East	258	28 (10.9)	152 (58.9)	59 (22.9)	19 (7.4)
Upper West	338	58 (17.2)	206 (61.0)	62 (18.3)	12 (3.6)
Volta	567	23 (4.0)	265 (46.0)	190 (33.0)	98 (17.0)
Western	638	16 (2.5)	262 (41.1)	204 (32.0)	156 (24.5)

MSLC^a^ Middle School Leaving Certificate

Social class^a^: Social class based on registrar general’s occupational classification England and wales of previous occupation during active service

The prevalence of obesity and overweight stratified by some common medical conditions and other medically related conditions is as shown in Table 3. The prevalence of hypertension was higher among known hypertensive patients (23.0%) compared to non-hypertensive patients (10.4%). Similarly there was a higher prevalence of obesity among known diabetics (24.6%) compared to non-diabetics (15.2%). Also with regards to total serum cholesterol measurement, pensioners with total serum cholesterol lower or equal to 6.18 mmol/L (239 mg/dL) had lower prevalence of obesity (14.7%) compared to pensioners with total serum cholesterol greater than 6.18 mmol/L (239 mg/dL) where the prevalence of obesity was 25.7%. The prevalence of obesity and overweight is lower among alcoholics and smokers.

**Table 3:** Prevalence of underweight, normal, overweight and Obesity among pensioners in Ghana, stratified by some medical and other medical related factors

		Body Mass index groupings			
		≤18.4	18.5–24.9	25.0–29.9	≥ 30
		Underweight	Normal	Overweight	Obese
Variable	*N*	*n* (%)	*n* (%)	*n* (%)	*n* (%)
Hypertensive Status					
Not Known/Normal	2267	179 (7.9)	1286 (56.7)	567 (25.0)	235 (10.4)
Known Hypertensive	2063	77 (3.7)	782 (37.9)	730 (35.4)	474 (23.0)
Diabetes Status					
Not known/ Normal	3818	249 (6.5)	1896 (49.7)	1092 (28.6)	581 (15.2)
Known Diabetic	435	6 (1.4)	143 (32.9)	179 (41.2)	107 (24.6)
Total serum cholesterol level (mmol/L)					
≤ 6.18 (239 mg/dL)	3813	246 (6.5)	1887(49.5)	1121 (29.4)	559 (14.7)
> 6.18 (239 mg/dL)	613	20(3.3)	226 (36.9)	209 (34.1)	158 (25.7)
Smoking status					
Non Smoker	4061	224 (5.5)	1922 (47.3)	1239 (30.8)	666 (16.4)
Smoker	93	13 (14.0)	61 (65.6)	11 (11.8)	8 (8.6)
Alcohol intake					
No Alcohol	3140	154 (4.9)	1431 (45.6)	988 (31.5)	567 (18.1)
Takes Alcohol	1031	82 (8.0)	557 (54.0)	284 (27.6)	108 (10.5)
Vegetarian status					
Not a vegetarian	4041	220 (5.4)	1920 (47.5)	1237 (30.6)	664 (16.4)
Vegetarian	93	12 (12.9)	49 (52.7)	26 (28.0)	6 (6.5)
Use of eye glasses/lenses					
No eye glasses	1826	153 (8.4)	1031 (56.5)	468 (25.6)	174 (9.5)
Uses eye lenses	2327	79 (3.4)	952 (40.9)	793 (34.1)	503 (21.6)
Diagnosed as having Arthritis					
No arthritis	3403	204 (6.0)	1709 (50.2)	1001 (29.4)	489 (14.4)
Have arthritis	691	40 (5.8)	272 (39.4)	219 (31.7)	160 (23.2)

### Factors associated with obesity and overweight among the pensioners in Ghana

Table 4 displays the crude odd ratio of logistic regression. Females were about four times (OR = 4.1, 95% CI 3.6–4.7) more likely to be obese/overweight. There was a gradual decrease in the odds of being obese /overweight with increasing age. Age range 65–69 years had an odds ratio of 0.7 (95% CI 0.6–0.9) whiles age group 80 and above was found to be about 70% protective from being overweight or obese (OR = 0.3, 95% CI = 0.2–0.5). The professional social class made up previous occupation of nurses, doctors, lawyers etc., whilst in active service, had the highest odds of being overweight or obese with an odds ratio of 5.6 (95% CI 4.2–7.6).

**Table 4:** Crude odds ratio of independent socio-demographic factors associated with overweight and obesity among pensioners in Ghana

Variable	*N* (%)	Crude Odds ratio	95% Confidence interval
Sex			
Male	3300 (69.0)	-	-
Female	1482 (31.0)	4.1	3.6–4.7
Age			
Less than 65	1664 (37.3)	-	-
65–69	1519 (34.1)	0.7	0.6–0.9
70–74	793 (17.8)	0.5	0.4–0.6
75–79	345 (7.7)	0.4	0.3–0.5
≥ 80	137 (3.1)	0.3	0.2–0.5
Social class			
I [Professional]	313 (7.1)	5.6	4.2–7.6
II [Managerial/technical]	799 (18.1)	1.9	1.6–2.4
III [(N) Skilled non-manual]	1333 (30.0)	2.1	1.7–2.6
III [(M) Skilled Manual]	402 (9.1)	1.2	0.9–1.5
IV [Partly skilled]	885 (20.0)	1.4	1.2–1.8
V [Unskilled]	694 (15.7)	-	-
Current Marital status			
Never Married	76 (1.7)	-	-
Married	3238 (73.1)	0.8	05–1.2
Widow/Widower	712 (16.1)	1.5	0.9–2.4
Divorced	220 (5.0)	1.5	0.9–2.6
Separated	182 (4.1)	1.2	0.7–2.1
Highest Formal Educational Level			
None	493 (12.0)	-	-
Basic/ MSLC^#^	1634 (39.8)	1.8	1.4–2.3
Secondary	630 (15.4)	2.0	1.6–2.7
Tertiary	1177 (28.7)	3.0	2.4–3.8
Others	168 (4.1)	2.7	1.9–3.9
Ethnicity			
Ga-Dangme	188 (3.9)	2.1	1.4–3.0
Akan	2283 (47.4)	1.6	1.3–2.0
Ewe	763 (15.9)	1.5	1.2–1.9
Guan	100 (2.1)	1.5	1.0–2.3
Mole-Dagomba	846 (17.6)	0.7	0.5–0.9
Other ethnic groups	633 (13.2)	-	-
Religion			
Christianity	3894 (86.8)	0.9	0.3–2.4
Islam	480 (10.7)	0.6	0.2–1.5
Traditional	94 (2.1)	0.1	0.0–0.3
Others	19 (0.4)	-	-

MSLC^#^ Middle School Leaving Certificate

With respect to marital status, being widowed/widower or divorced was associated with the highest odds of being obese/overweight compared to the other marital status. Being widow/widower was associated with an odds ratio of 1.5 (95% CI = 0.9–2.4). Being divorced is also associated with an odds ratio of 1.5 (95% CI = 0.9–2.6). With respect to highest educational level, the study found out that, the higher the educational level, the higher the odds of being obese/overweight. Pensioners who were educated up to the tertiary level had an odds ratio 3.0 (95% CI = 2.4–3.8) of being obese/overweight. Across the various ethnic groupings in Ghana, being a Ga-Dangme was associated with the highest odds of being obese/overweight (OR = 2.1, 95% CI = 1.4–3.0).

Table 5 displays the crude odds ratios and 95% confidence interval for the association of obesity/overweight with the medically related factors. Hypertensive pensioners had 2.6 times the odds of being obese/overweight compared to non-hypertensive pensioners (95% CI = 2.3–2.9). Diabetics were associated with higher odds of being obese/overweight, OR = 2.5 (95% CI = 2.0–2.0) compared to non-diabetics. Other factors associated with high odds of being overweight or obese includes; Serum triglyceride level 2.26 mmol/L (200 mg/dL) and above (OR = 2.6, 95% CI = 2.0–3.3), the use of eye glasses to aid vision (OR = 2.3, 95% CI = 2.0–2.6), total serum cholesterol level above 6.18 mmol/L or 239 mg/dL (OR = 1.9, 95% CI = 1.6–2.3), History of arthritis (OR = 1.6, 95% CI = 1.3–1.8) and history of allergies (OR = 1.7, 95% CI = 1.4–2.0). Also vegetarianism was protective with an odds ratio of 0.6 (95% CI = 0.4–0.9).

**Table 5:** Table showing Crude odds ratio of independent medically related factors associated with overweight and obesity among pensioners in Ghana

Variable	*N* (%)	Crude Odds ratio	95% Confidence interval
Known Hypertension Status			
Non Hypertensive	2317 (52.2)	-	-
Known Hypertensive	2122 (47.8)	2.6	2.3–2.9
Known Diabetic status	3914 (89.7)		
Not a known Diabetic	448 (10.3)	-	-
Known Diabetic		2.5	2.0–3.0
Use of eye glasses to aid vision			
No eye glass	1879 (44.1)	-	-
Uses eye glasses	2379 (55.9)	2.3	2.0–2.6
Diagnosed as having Arthritis			
No arthritis	3490 (83.1)	-	-
Have arthritis	710 (16.9)	1.6	1.3–1.8
Chronic bodily pains			
No bodily Pains	2154 (51.2)	-	-
Chronic bodily pains	2055 (48.8)	1.3	1.1–1.5
Allergies			
No Allergies	3583 (87.4)	-	-
Allergies	516 (12.6)	1.7	1.4–2.0
Previous surgeries			
No previous surgeries	2967 (70.3)		
Previous surgeries	1254 (29.7)	1.3	1.2–1.5
Alcohol intake			
No alcohol	3214 (75.2)	-	-
Takes in Alcohol	1062 (24.8)	0.6	0.5–0.7
Smoking status			
Non-Smoker	4162 (97.7)	-	-
Smoker	96 (2.3)	0.3	0.2–0.5
Mean Arterial Pressure (mmHg)			
≤ 105	2825 (61.9)	-	-
> 105	1736 (38.1)	1.4	1.3–1.6
Total Cholesterol level in mmol/L			
≤ 6.18 (239 mg/dL)	4076 (86.0)	-	
> 6.18 (239 mg/dL)	662 (14.0)	1.9	1.6–2.3
Low density Lipoprotein in mmol/L			
≤ 4.11 (159 mg/dL)	4187 (88.4)	-	-
≥ 4.12 (160 mg/dL)	551 (11.6)	1.5	1.3–1.8
Triglycerides level in mmol/L			
≤ 2.25 (199 mg/dL)	4449 (93.9)	-	
≥ 2.26 (200 mg/dL)	289 (6.1)	2.6	2.0–3.3
High density Lipoprotein in mmol/L			
< 1.55	2956 (62.4)	-	-
≥ 1.55	1782 (37.6)	1.4	1.2–1.6
Coronary risk ratio			
< 3.50		-	-
> 3.50		1.2	1.0–1.3
Hearing loss in one or both ears			
Normal hearing	4126 (95.1)	-	
Impaired hearing	213 (4.9)	0.6	0.5–0.9
Exercise lasting for at least 30 min in a day			
None per week	2924 (60.8)	-	-
1 or 2 days per week	707 (14.7)	1.4	1.2–1.7
3 days per week	271 (5.6)	1.1	0.9–1.4
> 3 days per week	911 (18.9)	1.0	0.9–1.2
Vegetarian status			
Non vegetarian	4143 (97.8)	-	-
Vegetarian	95 (2.2)	0.6	0.4–0.9

Table 6 displays the odds ratio, 95% confidence intervals and *p*-values obtained in three models. Adjusting for all other variables as found in model C, the female pensioners were associated with three times the odds of being obese/overweight compared to the male pensioners. Hypertensive pensioners had 1.8 times the odds (95% CI = 1.5–2.0) of being obese/overweight. Among the serum lipids, triglycerides levels of 2.26 mmol/L (200 mg/dL) or more, was most associated with obesity and overweight among the pensioners. Pensioners with serum triglyceride level of 2.26 mmol/L (200 mg/dL) were associated with 1.8 times the odds of being obese/overweight compared to those with triglyceride levels below 2.26 mmol/L (200 mg/dL). Arthritis was more common among the obese/overweight group compared to the normal/underweight pensioners. Also the use of eyeglasses in the elderly was associated with increased odds of obesity and overweight (OR = 1.7, 95% CI = 1.3–2.2).

**Table 6:** Adjusted odds ratio for factors independently associated with Obesity and overweight by models

	Model A	Model B	Model C
Independent factors	AOR (95% CI)	AOR (95% CI)	AOR (95% CI)
Known Hypertension Status			
Non Hypertensive	-		
Known Hypertensive	2.3 (2.1–2.7)	2.1 (1.8–2.4)	1.8 (1.5–2.0)
Known Diabetic status			
Not a known Diabetic	-		
Known Diabetic	2.3 (1.8–2.9)	2.0 (1.5–2.5)	1.7 (1.3–2.2)
Use of eye glasses to aid vision			
No eye glass	-		
Uses eye glasses	2.1 (1.8–2.4)	1.5 (1.3–1.8)	1.6 (1.4–1.9)
Mean Arterial Pressure (mmHg)			
≤ 105	-		
> 105	1.6 (1.4–1.9)	1.8 (1.5–2.1)	1.6 (1.3–1.9)
Diagnosed as having Arthritis			
No arthritis	-		
Have arthritis	1.3 (1.2–1.5)	1.4 (1.1–1.7)	1.3 (1.1–1.6)
Previous surgeries			
No previous surgeries	-		
Previous surgeries	1.3 (1.2–1.5)	1.3 (1.1–1.5)	1.2 (1.0–1.4)
Smoking status			
Non-Smoker	-		
Smoker	0.4 (0.2–0.7)	0.5 (0.3–0.9)	0.5 (0.3–1.0)
Hearing loss in one or both ears			
Normal hearing	-		
Impaired hearing	0.7 (0.5–0.9)	0.7 (0.5–0.9)	0.7 (0.5–1.0)
Total Cholesterol level in mmol/L			
≤ 6.18 (239 mg/dL)	-		
> 6.18 (239 mg/dL)	2.1 (1.7–2.6)	1.6 (1.3–2.0)	1.6 (1.1–2.2)
Triglycerides level in mmol/L			
≤ 2.25 (199 mg/dL)	-		
≥ 2.26 (200 mg/dL)	2.4 (1.8–3.2)	2.0 (1.5–2.7)	1.8 (1.3 - 2.5)
Low density Lipoprotein in mmol/L			
≤ 4.11 (159 mg/dL)	-		
≥ 4.12 (160 mg/dL)	1.7 (1.4–2.1)	1.3 (1.1–1.7)	
Coronary risk ratio			
< 3.50	-		
> 3.50	1.3 (1.2–1.5)	1.2 (1.0–1.4)	
Sex			
Male	-		
Female	4.3 (3.7–5.1)		3.1 (2.5–3.8)
Highest Formal Educational Level			
None	-		
Basic/ MSLC^#^	1.4 (1.0–1.8)		
Secondary	1.8 (1.4–2.4)		
Tertiary	2.5 (1.9–3.3)		
Others	1.8 (1.2–2.7)		
Social class^#^			
I [Professional]	4.6 (3.3–6.3)		
II [Managerial/technical]	1.6 (1.3–2.0)		
III [(N) Skilled non-manual]	1.8 (1.4–2.2)		
III [(M) Skilled Manual]	1.1 (0.8–1.4)		
IV [Partly skilled]	1.2 (1.0–1.5)		
V [Unskilled]	-		

MSLC^#^ Middle School Leaving Certificate

Model A: Model Adjusted for religion, region of residence, ethnic background and marital status

Model B: Model adjusted for age, sex, Highest formal education, Social class and all the variables adjusted for in model A

Model C: Model adjusted for all variables adjusted for in model A, plus all other variables in the table

Reporting for variables with significant results in model C

## Discussion

### Prevalence of obesity

The high prevalence of obesity among females compared to males have been well documented and consistently found in many studies involving African and African-American women [23, 24]. The number of females in the study was low compared to the males. This is contrary to what was reported in the 2010 census, special report on the elderly where 51.2% were females and 48.8% were males [8]. Majority of the women in Ghana works in the informal sector and many end up as house wives with no formal social security or pension scheme. Most of them do not form part of the national pensioners association [25]. We believe the women in our study had a higher social class and economic status compared to the average Ghanaian elderly woman. This we believe contributed to the exaggerated difference in the prevalence of obesity among the female and male pensioners in Ghana. Also, the established higher percentage body fat in women compare to men may play a part [26].

### Factors associated with obesity

With regards to factors associated with obesity and overweight, the study found out that age has an inverse relationship with BMI. Obesity and overweight are associated with high morbidity [13, 27] and mortality and as such the obese individuals were more likely to have died leaving low prevalence of obesity across the age groups. Some studies actually found excess death associated with obesity and overweight in the elderly population [28].

Physical exercise 30 min or more three times per-week was associated with obesity in the elderly. It could be explained that the proportion of the elderly likely to be currently involved in routine physical exercise were those that were obese aiming at weight reduction. Secondly, physical exercise by the elderly is likely to be grossly limited by diseases associated with old age. Smoking appears to be protective for obesity among the pensioners, it could be explained that, smokers were more at risk of malnutrition and underweight. Also, smokers were at risk to cancers and certain diseases conditions that can result in wasting and hence the impression of smoking being protective against obesity/overweight.

We observed significant variations across the regions and ethnic groups in the country. The greater Accra region of Ghana had the highest prevalence of obesity whiles the upper west region had the least prevalence of Obesity. Accra, the capital of Ghana located within the greater Accra region has the most infrastructural and economic development in the country. Socio-economic variation across the regions may play a role in the observed variation in Obesity. Also, among the ethnic groups, the Ga-Dangme people lives within the greater Accra region. Socio-economic factors, lifestyle and family eating habits could play a role in the observed ethnic variations across the country.

Currently there are programs within the Ghana health service seeking to address the growing challenge of obesity and overweight in the country. The ‘good life” initiative of the ministry of health, Ghana health service and its partners seeks among other things to promote healthy lifestyle, responsible eating and exercise which has the potential to help halt the increasing trend of obesity and overweight in the country.

This study suffers some limitations. In the 2010 population and housing report on the elderly in Ghana [8], it was found out that the female elderly population was 56% as oppose to 44% of males which was attributed to higher life expectancy of females than males. However in this study, males constituted 68.3% while females constituted 31.7% of pensioners. This could be explained that, majority of females work in the informal sector of the country and are not members of the national pensioners association. Hence the low proportion of females surveyed in this study, which could introduce a bias when generalizing the results of the study on the pensioners to include the whole elderly population of Ghana. Despite the above limitations, we believe the study has implications in informing evidence based policy formulation and decision making regarding obesity and overweight in Ghana. It will also influence public health intervention on priority areas or regions where interventions are necessary to halt the rising trend of obesity and overweight in the country.

## Conclusion

In conclusion, the prevalence of obesity and overweight among the pensioners is high. Hypertension, Diabetes, dyslipidemia, use of medicated glasses, female sex and professional social class whilst in active service were significantly associated with obesity and overweight. We recommend that the Ghana Health service institute active behavioral change communication and health promotion strategies among the youth and young adults with regard to maintaining an ideal weight so as to reduce the prevalence of obesity and overweight, which is likely to continue in pension life, if not addressed early.

## Abbreviation

BMI: Body Mass Index
GHS: Ghana Health Service
NPA: National Pensioners Association
PMS: Pensions Medical Scheme
